# Modeling Left Ventricle Perfusion in Healthy and Stenotic Conditions

**DOI:** 10.3390/bioengineering8050064

**Published:** 2021-05-11

**Authors:** Marilena Pannone

**Affiliations:** School of Engineering, University of Basilicata, 85100 Potenza, Italy; marilena.pannone@unibas.it

**Keywords:** blood flow, hemodynamics, left ventricle perfusion, deterministic fluid-mechanical model, computational fluid-dynamics, ischemic conditions, in vivo observations validation

## Abstract

A theoretical fluid mechanical model is proposed for the investigation of myocardial perfusion in healthy and stenotic conditions. The model hinges on Terzaghi’s consolidation theory and reformulates the related unsteady flow equation for the simulation of the swelling–drainage alternation characterizing the diastolic–systolic phases. When compared with the outcome of experimental in vivo observations in terms of left ventricle transmural perfusion ratio (T.P.R.), the analytical solution provided by the present study for the time-dependent blood pressure and flow rate across the ventricle wall proves to consistently reproduce the basic mechanisms of both healthy and ischemic perfusion. Therefore, it could constitute a useful interpretative support to improve the comprehension of the basic hemodynamic mechanisms leading to the most common cardiac diseases. Additionally, it could represent the mathematical basis for the application of inverse methods aimed at estimating the characteristic parameters of ischemic perfusion (i.e., location and severity of coronary stenoses) via downstream ventricular measurements, possibly inspiring their assessment via non-invasive myocardial imaging techniques.

## 1. Introduction

The factors that are usually responsible for triggering ischemic heart diseases can be identified in the reduction in coronary reserve below a lower limit, and/or in the increase in myocardial oxygen consumption above an upper physiological threshold. An arteriosclerotic lesion located within an epicardial vessel determines a downstream pressure drop that increases with decreasing vessel’s free diameter. The pressure gradient arising from the constriction of the resistance conduit induces an upstream dilatation that is normally sufficient to keep an adequate myocardial blood inflow in basal conditions. However, if stenosis was severe, even the basal flux would be compromised and the entire coronary tree should employ most of its reserve to convey the minimum vital metabolic supply towards the heart muscle. Thus, in the simultaneous presence of severe coronary stenosis and increased metabolic demand, the coronary circuit may be not able to satisfy it anymore leading to the so-called myocardial ischemia. When the interruption of blood flux is almost complete, and in the absence of lateral circulation, the necrosis of the tissue will affect the whole ventricle thickness (myocardial transmural infarction). Conversely, if the thrombus determines a transient occlusion (less than 3 h) or a subocclusive stenosis in the presence of considerable lateral circulation, the necrosis will usually be confined to the subendocardial layers [1]. In the recent past, many research papers and reviews in literature have discussed the relationship between perfusion abnormalities due to distal coronary stenosis and myocardial blood flow [2,3], the differences between subendocardial and transmural infarction [4,5,6], their possible causes and the most suitable biomedical techniques aimed at detecting and analyzing them [7,8,9,10,11,12,13,14,15,16,17,18,19]. The present study proposes a theoretical analogical model able to grasp the main time-dependent fluid-mechanical features of myocardial wall perfusion in normal conditions and in the presence of coronary occlusions that determine a reduced blood inflow towards a more or less extended ventricle sector. The model provides pressure and flow rate distribution in the ventricular large- and medium-scale arterial network and accounts for the microscale circulation (capillary flow) by a suitable sink term. The effect of a possible altered metabolic demand is included via the time-variation of the capillary transfer rate. The resulting pattern of pathological isobars and isotachs identifies the contours of the potential necrotic areas in the case of undisturbed disease progression. The model hinges on porous media consolidation theory by adapting the corresponding unsteady flow equation to the biological district of interest, which is characterized by an eminently hierarchical vascular organization and external periodic recharge. The complex phenomenon of ventricle wall perfusion as a function of healthy and stenotic coronary blood supply is modelled in the attempt of providing a possible interpretative support for the common cardiovascular medical therapies. Additionally, since the proposed model is fully analytical and yields an exact solution for the local time-dependent myocardial pressure and flow rate, it may be particularly useful for the application of inverse methods [20,21] aimed at estimating the ischemic perfusion parameters that better fit downstream ventricular measurements. Thus, it could inspire the detection of exact location and severity of coronary stenoses via myocardial imaging techniques [22,23,24,25,26,27].

## 2. Methods

**Figure 1:**
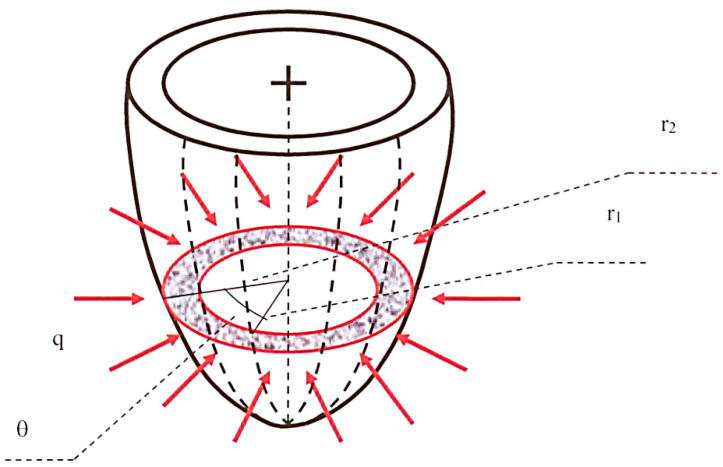
Sketch of the left ventricle model.

The fluid-mechanical analogical model adopted in the present study to simulate the left ventricular wall and its physiopathological function consists of a porous semiellipsoidal shell (see also van Den Broek and van Den Broek [28]) characterized by radially decreasing permeability, which should be seen as the continuous equivalent of the muscular myocardial fibers penetrated towards the internal cavity by decreasing average-diameter vessels. The supplying coronary arteries and the collecting veins, from which the intra-myocardial vessels originate or in which they end, develop along the directrices of the external shell surface. Figure 1 shows a simplified scheme of this model. The grey annulus with red contours represents the generic short-axis section of the ventricle wall, the red arrows indicate coronary blood inflow at rate *q* = *q*(*t*) per unit ventricle height, and the dotted lines schematize the epicardial supplying/collecting vessels.

Arterioles and venules, which jointly constitute the pores of the equivalent saturated porous medium, are assimilated to two symmetrical and physically intertwined hierarchical networks, communicating with each other by the capillaries and connected in series at the endocardial boundary. The arterial network receives blood at the epicardium and carries it toward the endocardium, that is, from the external to the internal shell surface. At each level along the radial direction, the hydraulic and the osmotic gradient allow for the exchange of oxygen and nutrients between the arterial branch of the capillaries and the tissue. The venous network, in turn, collects blood deprived of oxygen from the endocardium to the epicardium, that is, from the internal to the external shell surface. At each level along the radial direction, the hydraulic and the osmotic gradient allow for the exchange of carbon dioxide between the tissue and the venous branch of the capillaries. It must be emphasized that, in the present 2-D analytical approach, only the arterial network function will be simulated with reference to a unit-height, approximately cylindrical and horizontal short-axis slice of the ventricle, which will be assumed as structurally homogeneous in the vertical direction. The blood entering it at the epicardial boundary diffuses through radially decreasing average-diameter vessels; the portion of fluid that gradually flows into the capillaries, and subsequently into the venous network, practically leaves the system. At endocardium (conventionally coinciding with the arterial/venous limit of the capillaries at the purely capillary level of the coronary hierarchy) the blood wave is exhausted, the flow reverses its direction, and the pressure (*p*) can be considered as time-invariant [29]. In the porous media framework, this schematization translates into an unsteady and dissipative fluid flow governed by an axial-symmetric hydraulic conductivity (*K*) that increases with the radial distance from the center of the ventricle lumen. In the present basic version of the model, the en bloc motion of the ventricle wall, its periodic twisting and the systolic/diastolic variation of its thickness are neglected. The horizontal elastic deformation of vessels and myocardial fibers is incorporated in the analytical solution via the specific storage (*S_s_*) of the equivalent saturated porous medium. The specific storage mathematically allows for the swelling-drainage alternation characterizing the diastolic-systolic phases as a result of the internal rearrangement of the areas occupied by solid and fluid component. The periodicity of the coronary inflow determined by the contraction of the heart muscle is accounted for by an external-boundary harmonic blood recharge. Finally, the arterial–venous network exchange is simulated by a suitable sink term.

Terzaghi’s theory of soil consolidation [30] assumes that a constant external overload σ applied to a saturated aquifer in the vertical direction is absorbed in part by the solid component (increase in effective stress $\bar{\sigma }$) and in part by the interstitial water (increase in fluid pressure *p*): $\sigma =\bar{\sigma }+p$. Due to fluid pressure increment, a transient flow is started, the water is drained, *p* decays and the effective stress is further increased causing compaction. In the case of ventricle perfusion, we will assume that:

- The solid phase is represented by myocardial fibers and walls of vessels;
- The interstitial fluid is represented by the blood filling the myocardial vessels;
- The arterial network is responsible for the diastolic circumferential swelling process;
- The external overloading consists in a rhythmic vertical contraction of the heart muscle;
- $\bar{\sigma }$ indicates the vertical stress absorbed by the muscular fibers;
- *p* is the so-called transmural pressure (i.e., the isotropic blood pressure within the vessels that exceeds the compression transmitted by the muscular fibers);
- σ is the total vertical stress within the ventricle wall;
- The systolic circumferential drainage takes place through the venous network.

During diastole, the muscular fibers relax and the vessels dilate due to coronary perfusion: $\bar{\sigma }$ decreases and *p* increases. Conversely, during systole, the muscular fibers contract and the vessels collapse due to the gradual interruption of coronary inflow: $\bar{\sigma }$ increases and *p* decreases. At the peak of diastole, the muscles are completely relaxed, the vessels are filled with blood and:

(1) σ≅p

At the peak of systole, there is no blood flux towards the ventricle, the whole blood volume has been squeezed out by the contracted myocardium and

(2) $$\sigma ≅\bar{\sigma }$$

The combination of Darcy’s law U=−(K/γ)∇p (where **U** indicates flow rate vector across the two-component medium and *γ* fluid specific weight), equation of continuity for solid and fluid component, and the constitutive relation of the fluid, leads to the fluid transient diffusion equation [30], here expressed in local cylindrical coordinates (*r*, *θ*, *z*) in the absence of considerable vertical blood flux ($\partial^{2}p/\partial z^{2}≅0$):

(3) $$S_{s}\frac{\partial p}{\partial t}=\frac{1}{r}\left[\frac{\partial }{\partial r}\left(rK\frac{\partial p}{\partial r}\right)\right]+\frac{K}{r^{2}}\frac{\partial^{2}p}{\partial \theta^{2}}-\gamma \tilde{q}$$

In Equation (3) and in what follows, $\tilde{q}$ indicates the volume of blood leaving the arterial network via the capillaries per unit time and per unit ventricle volume, and *r* and *θ* the radial and the angular coordinate, respectively (Figure 1). Additionally, the specific storage is given by:

(4) $$S_{s}=\gamma \omega \left[\beta_{f}+\frac{\left(\omega -1\right)}{\omega }\varepsilon_{s}+\frac{\varepsilon }{\omega }\right]$$

where *ω* represents porous medium porosity, *ε* the bulk coefficient of elasticity, *ε_s_* the coefficient of elasticity of the muscular component, *β_f_* the coefficient of compressibility of blood and [30]:

(5) $$K=C\frac{\gamma }{\mu }d_{v}^{2}=Dd_{v}^{2}$$

In Equation (5), *C* is a coefficient depending on pore/vessel number and geometry, *d_v_* = *d_v_*(*r*) indicates pore/vessel average diameter, *μ* blood viscosity and *D* = *Cγ*/*μ*.

For fixed blood density and viscosity, the conductivity, specific storage and capillary transfer rate must be directly proportional to porosity (i.e., to the percentage of ventricle wall volume instantaneously occupied by blood-filled vessels). In the present study, all the solutions of the governing Equation (3) were obtained by assuming that the increase/reduction in *S_s_*(*ω*(*t*)) was matched by a proportionate increase/reduction in *D*(*ω*(*t*)) and $\tilde{q}\left(\omega \left(t\right)\right)$. Additionally, it was assumed that *d_v_* varies linearly with the radial distance *r* along the vessel tree. According to these hypotheses, the ratios *D*/*S_s_* and $\tilde{q}/S_{s}$ were conventionally evaluated in average conditions (mid diastole/mid systole) and considered as constants for each given perfusion condition, obtaining:

(6) $$\gamma \tilde{q}/S_{s}=\mathrm{const}=\delta$$

and

(7) $$\frac{K}{S_{s}}=\mathrm{const}\times r^{2}=\frac{K_{1}}{S_{s1}r_{1}^{2}}r^{2}=\alpha r^{2}$$

where subscript 1 refers to the endocardial boundary.

## 3. Results

### 3.1. Solution for Homogeneous Perfusion

**Figure 2:**
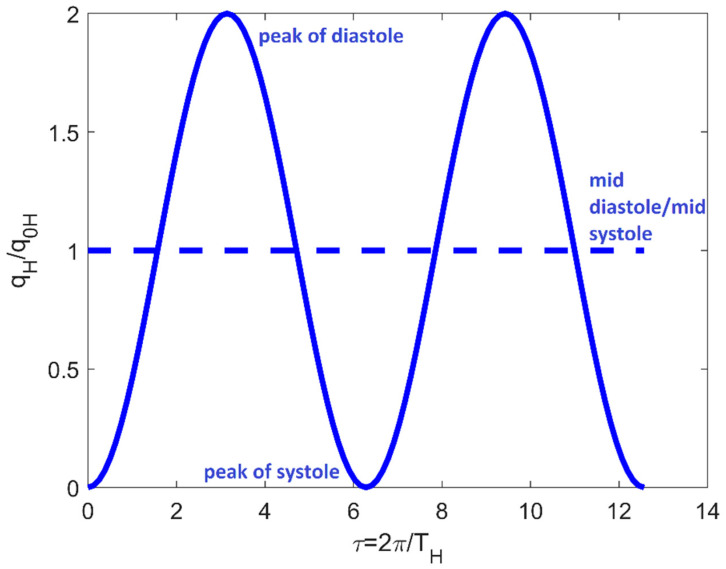
Time-dependent boundary condition in system (8). Full line is referred to the periodic inflow rate; dotted line indicates the mid-diastole/mid systole value.

The adopted fluid-mechanical model of myocardial wall perfusion, and the condition of healthy axial-symmetric inflow at epicardium, lead to the following boundary value problem:

(8) $$\left\{ \begin{array}{l} \frac{\partial p_{H}}{\partial t}=\frac{1}{r}\left[\frac{\partial }{\partial r}\left(\alpha r^{3}\frac{\partial p_{H}}{\partial r}\right)\right]-\delta_{H}\\ p_{H}\left(r,0\right)=0\\ p_{H}\left(r_{1},t\right)=0\\ {\frac{\partial p_{H}}{\partial r}|}_{\left(r_{2},t\right)}=\frac{\gamma q_{H}\left(t\right)}{2\pi r_{2}K_{2}} \end{array}\right.r_{1}\leq r\leq r_{2}\;\;t\geq 0$$

where subscript *H* stands for healthy-control conditions, *p_H_* is the healthy transmural pressure exceeding the constant value that is measured at the arterial–venous limit of the capillaries (*p_c_* ≅ 20 mmHg), *K*_2_ = *K*(*r*_2_), $r_{2}=r_{1}+s$ and *s* indicates ventricle thickness (8 mm on average [31]). Additionally,

(9) $$q_{H}\left(t\right)=q_{0}{}_{H}f\left(t\right)=q_{0H}\left[1-\cos\left(\frac{2\pi t}{T_{H}}\right)\right]$$

represents the leading-harmonics approximation of the pulsatile inflow rate per unit ventricle height, *q*_0*H*_ indicates the corresponding mid diastole/mid systole value and *T_H_* is the healthy-control cardiac cycle period (Figure 2).

Note that the baseline healthy case should represent not only ventricular perfusion at rest in the absence of coronary occlusions, but also stenotic perfusion when efficient mechanisms of autoregulation can still be triggered.

For the handling of the time-dependent boundary condition, the solution of system (8) was pursued by applying Duhamel’s convolution [32]:

(10) $$p_{H}\left(r,t\right)=\int_{0}^{t}p_{H}^{*}\left(r,t-\tau \right)\frac{df\left(\tau \right)}{d\tau }d\tau +p_{H}^{*}\left(r,t\right)f\left(0\right)$$

where $p_{H}^{*}$ is the solution of the problem for time-invariant boundary conditions ${\partial p/\partial r|}_{\left(r_{2},t\right)}=\gamma q_{0H}/2\pi r_{2}k_{2}$. The boundary value problem for $p_{H}^{*}$ was solved based on Sturm–Liouville’s algorithm and Fourier’s theorem [33]. Fourier decomposition was already used by the author to solve deterministic and stochastic advection–diffusion equations in bounded Cartesian spaces, with time-invariant boundary conditions and uniform or space-dependent coefficients [34,35,36].

The initial condition assumed in system (8) corresponds to the start-up of the system, which is necessarily characterized by an idealized situation of no flux. Healthy ventricle perfusion will therefore be represented by the regime version of the general result (*t* → ∞):

(11) $$\begin{array}{l} p_{H}\left(r,t\right)=\left\{\frac{\delta_{H}}{3\alpha }\ln\left(\frac{r}{r_{1}}\right)+\left(\frac{\gamma q_{0H}}{6\pi K_{2}}-\frac{\delta_{H}}{9\alpha }\right)\left[{\left(\frac{r_{2}}{r_{1}}\right)}^{3}-{\left(\frac{r_{2}}{r}\right)}^{3}\right]\right\}\left[1-\cos\left(\frac{2\pi t}{T_{H}}\right)\right]+\frac{2\pi }{T_{H}}{\left(\frac{r_{1}}{r}\right)}^{3/2}\cdot \\ \sum_{m=1}^{\infty }b_{m}\left[\frac{\Omega_{m}\sin\left(\frac{2\pi t}{T_{H}}\right)-\frac{2\pi }{T_{H}}\cos\left(\frac{2\pi t}{T_{H}}\right)}{\Omega_{m}^{2}+4\pi^{2}/T_{H}^{2}}\right]\sin\left[\frac{\sqrt{\nu_{m}}}{W}\ln\left(\frac{r}{r_{1}}\right)\right] \end{array}$$

where:

(12) $$\begin{array}{l} b_{m}=-\frac{2\delta_{H}W}{3\alpha \left(\frac{9}{4}W^{2}+\nu_{m}\right)}\{\exp\left(\frac{3}{2}W\right)[\left(\frac{3}{2}W-\frac{\frac{9}{4}W^{2}-\nu_{m}}{\frac{9}{4}W^{2}+\nu_{m}}\right)\sin\left(\sqrt{\nu_{m}}\right)-\\ \left(\sqrt{\nu_{m}}-\frac{3W\sqrt{\nu_{m}}}{\frac{9}{4}W^{2}+\nu_{m}}\right)\cos\left(\sqrt{\nu_{m}}\right)]-\frac{3W\sqrt{\nu_{m}}}{\frac{9}{4}W^{2}+\nu_{m}}\}+\frac{2\left(\frac{\gamma q_{0H}}{6\pi K_{2}}-\frac{\delta_{H}}{9\alpha }\right)}{\frac{9}{4}W^{2}+\nu_{m}}\{\exp\left(\frac{3}{2}W\right)[-\frac{3}{2}W\sin\left(\sqrt{\nu_{m}}\right)-\\ \sqrt{\nu_{m}}\cos\left(\sqrt{\nu_{m}}\right)]-\exp\left(\frac{9}{2}W\right)\left[\frac{3}{2}W\sin\left(\sqrt{\nu_{m}}\right)-\sqrt{\nu_{m}}\cos\left(\sqrt{\nu_{m}}\right)\right]\} \end{array}$$

(13) $$\Omega_{m}=\alpha \left(\frac{\nu_{m}}{W^{2}}+\frac{9}{4}\right)$$

(14) $$W=\ln\left(\frac{r_{2}}{r_{1}}\right)$$

and *ν_m_* is the mth solution of the trigonometric equation:

(15) $$\tan\left(\sqrt{\nu }\right)=\frac{2}{3W}\sqrt{\nu }$$

### 3.2. Solution for Non-Homogeneous Perfusion

Full or partial coronary stenosis induces the presence of a specific under-perfused ventricle sector. In these conditions, the suitable boundary value problem to be solved, which is not axial-symmetric anymore, can be formulated as follows in a time reference frame originating at the instant *t*_0_ of the generic cardiac cycle (0 ÷ *T*) when the thrombus forms, with the governing equations that now includes a circumferentially diffusive term:

(16) $$\left\{ \begin{array}{l} \frac{\partial p}{\partial t}=\frac{1}{r}\left[\frac{\partial }{\partial r}\left(\alpha r^{3}\frac{\partial p}{\partial r}\right)\right]+\alpha \frac{\partial^{2}p}{\partial \theta^{2}}-\delta \\ p\left(r,\theta ,0\right)=p_{H}\left(r,t_{0}\right)\\ p\left(r_{1},\theta ,t\right)=0\\ {\frac{\partial p}{\partial r}|}_{\left(r_{2},\theta ,t\right)}=\frac{\gamma q_{s}\left(\theta ,t\right)}{2\pi r_{2}K_{2}}\\ {\frac{\partial p}{\partial \theta }|}_{\left(r,0,t\right)}={\frac{\partial p}{\partial \theta }|}_{\left(r,\pi ,t\right)}=0 \end{array}\right.r_{1}\leq r\leq r_{2}\;0\leq \theta \leq 2\pi \;t\geq 0$$

**Figure 3:**
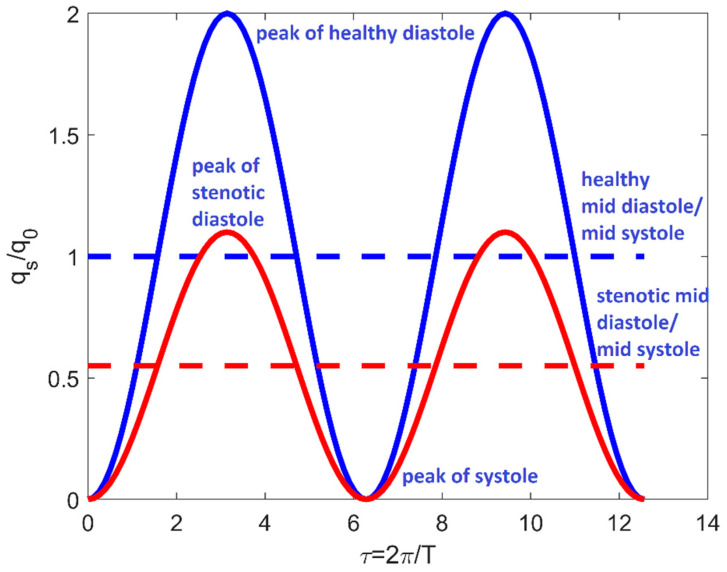
Time-dependent boundary condition in system (16) for *ξ* = 0.55 and *t*_0_ = 0. Red lines refer to −Θ*_s_*/2 ≤ θ < Θ*_s_*/2; blue lines refer to Θ*_s_*/2 ≤ *θ* < 2 π−Θ*_s_*/2. Full lines are referred to the periodic inflow rate; dotted lines indicate the mid-diastole/mid-systole value.

In (16), the stenotic inflow rate *q_s_* (*θ*, *t*) (Figure 3) is given by:

(17) $$q_{s}\left(\theta ,t\right)=\left\{ \begin{array}{l} q_{0}^{\prime }h\left(t\right)\;\;-\Theta_{s}/2\leq \theta <\Theta_{s}/2\\ q_{0}h\left(t\right)\;\;\Theta_{s}/2\leq \theta <2\pi -\Theta_{s}/2 \end{array}\right.$$

with $\Theta_{s}$ indicating the angle subtended by the ventricle sector affected by reduced perfusion,

(18) $$q_{0}^{\prime }=\xi q_{0}\;\;0\leq \xi <1$$

and

(19) $$h\left(t\right)=1-\cos\left[\frac{2\pi \left(t+t_{0}\right)}{T}\right]$$

It should be emphasized that the last condition of system (16) combined with Equation (17) implies the symmetry of the solution with respect to the axis that passes through the center of the underperfused sector. Additionally, as modelled by (16), ischemic perfusion has to be intended as taking place without the activation of collateral feeding circuits.

Starting from the separation of the original boundary value problem into a free-evolution and a forced-evolution subsystem, and repeatedly applying Duhamel’s principle and Sturm–Liouville’s algorithm after suitable transformations of variables, one obtains:

(20) $$\begin{array}{l} p\left(r,\theta ,t\right)=\{\left[\frac{\delta }{3\alpha }+{\tilde{B}}_{0}{\left(\frac{r_{1}}{r}\right)}^{\left(3/2\right)}\right]\ln\left(\frac{r}{r_{1}}\right)+\frac{\delta }{9\alpha }\left[{\left(\frac{r_{2}}{r}\right)}^{3}-{\left(\frac{r_{2}}{r_{1}}\right)}^{3}\right]+\\ \sum_{n=1}^{\infty }{\tilde{B}}_{n}\sinh\left[n\ln\left(\frac{r}{r_{1}}\right)\right]\cos\left(n\theta \right){\left(\frac{r_{1}}{r}\right)}^{\left(3/2\right)}\}h\left(t\right)+\sum_{m=1}^{\infty }B_{m}\left\{\Gamma_{m}\left(t\right)+\exp\left[-\alpha \left(\frac{\nu_{m}}{W^{2}}+\frac{9}{4}\right)t\right]h\left(0\right)\right\}\cdot \\ \sin\left[\frac{\sqrt{\nu_{m}}}{W}\ln\left(\frac{r}{r_{1}}\right)\right]{\left(\frac{r_{1}}{r}\right)}^{\left(3/2\right)}+\sum_{n=1}^{\infty }\sum_{m=1}^{\infty }B_{mn}\left\{\Gamma_{mn}\left(t\right)+\exp\left[-\alpha \left(n^{2}+\frac{\nu_{m}}{W^{2}}+\frac{9}{4}\right)t\right]h\left(0\right)\right\}\cdot \\ \sin\left[\frac{\sqrt{\nu_{m}}}{W}\ln\left(\frac{r}{r_{1}}\right)\right]{\left(\frac{r_{1}}{r}\right)}^{\left(3/2\right)}\cos\left(n\theta \right) \end{array}$$

where:

(21) $$\begin{array}{l} B_{m}=\frac{\frac{2W}{3\alpha }\left\{\delta_{H}\left[1-\cos\left(\frac{2\pi t_{0}}{T_{H}}\right)\right]-\delta \right\}}{\frac{9}{4}W^{2}+\nu_{m}}\cdot \\ \{\exp\left(\frac{3W}{2}\right)\left[\left(\frac{3W}{2}-\frac{\frac{9}{4}W^{2}-\nu_{m}}{\frac{9}{4}W^{2}+\nu_{m}}\right)\sin\left(\sqrt{\nu_{m}}\right)-\left(\sqrt{\nu_{m}}-\frac{3W\sqrt{\nu_{m}}}{\frac{9}{4}W^{2}+\nu_{m}}\right)\cos\left(\sqrt{\nu_{m}}\right)\right]-\frac{3W\sqrt{\nu_{m}}}{\frac{9}{4}W^{2}+\nu_{m}}\}+\\ \frac{2\left\{\left(\frac{\delta_{H}}{9\alpha }-\frac{\gamma q_{0H}}{6\pi K_{2}}\right)\left[1-\cos\left(\frac{2\pi t_{0}}{T_{H}}\right)\right]-\frac{\delta_{H}}{9\alpha }\right\}}{\frac{9}{4}W^{2}+\nu_{m}}\{\exp\left(\frac{3W}{2}\right)\left[-\frac{3W}{2}\sin\left(\sqrt{\nu_{m}}\right)-\sqrt{\nu_{m}}\cos\left(\sqrt{\nu_{m}}\right)\right]-\\ \exp\left(\frac{9W}{2}\right)\left[\frac{3W}{2}\sin\left(\sqrt{\nu_{m}}\right)-\sqrt{\nu_{m}}\cos\left(\sqrt{\nu_{m}}\right)\right]\}+\frac{2\pi }{T_{H}}b_{m}\frac{\left[\Omega_{m}\sin\left(\frac{2\pi t_{0}}{T_{H}}\right)-\frac{2\pi }{T_{H}}\cos\left(\frac{2\pi t_{0}}{T_{H}}\right)\right]}{\Omega_{m}^{2}+4\pi^{2}/T_{H}^{2}}+\\ 2{\tilde{B}}_{0}W\left[\frac{\cos\left(\sqrt{\nu_{m}}\right)}{\sqrt{\nu_{m}}}-\frac{\sin\left(\sqrt{\nu_{m}}\right)}{\nu_{m}}\right] \end{array}$$

(22) $$B_{mn}=-\frac{2{\tilde{B}}_{n}}{n^{2}W^{2}+\nu_{m}}\left[nW\cosh\left(nW\right)\sin\left(\sqrt{\nu_{m}}\right)-\sqrt{\nu_{m}}\sinh\left(nW\right)\cos\left(\sqrt{\nu_{m}}\right)\right]$$

(23) $${\tilde{B}}_{0}=\frac{\gamma \exp\left(3W/2\right)}{2\pi^{2}k_{2}\left(1-3W/2\right)}\left[q\prime_{0}\frac{\Theta_{s}}{2}+q_{0}\left(\pi -\frac{\Theta_{s}}{2}\right)\right]$$

(24) $${\tilde{B}}_{n}=\frac{\gamma \exp\left(3W/2\right)\sin\left(n\Theta_{s}/2\right)\left(q\prime_{0}-q_{0}\right)}{n\pi^{2}k_{2}\left[n\cosh\left(nW\right)-\frac{3}{2}\sinh\left(nW\right)\right]}$$

(25) $$\begin{array}{l} \Gamma_{m}\left(t\right)=\frac{\frac{2\pi }{T}\cos\left(\frac{2\pi t_{0}}{T}\right)}{\Omega_{m}^{2}+4\pi^{2}/T^{2}}\left[\Omega_{m}\sin\left(\frac{2\pi t}{T}\right)-\frac{2\pi }{T}\cos\left(\frac{2\pi t}{T}\right)+\frac{2\pi }{T}\exp\left(-\Omega_{m}t\right)\right]+\\ \frac{\frac{2\pi }{T}\sin\left(\frac{2\pi t_{0}}{T}\right)}{\Omega_{m}^{2}+4\pi^{2}/T^{2}}\left[\Omega_{m}\cos\left(\frac{2\pi t}{T}\right)+\frac{2\pi }{T}\sin\left(\frac{2\pi t}{T}\right)-\Omega_{m}\exp\left(-\Omega \prime_{m}t\right)\right] \end{array}$$

(26) $$\begin{array}{l} \Gamma_{mn}\left(t\right)=\frac{\frac{2\pi }{T}\cos\left(\frac{2\pi t_{0}}{T}\right)}{\Omega_{mn}^{2}+4\pi^{2}/T^{2}}\left[\Omega_{mn}\sin\left(\frac{2\pi t}{T}\right)-\frac{2\pi }{T}\cos\left(\frac{2\pi t}{T}\right)+\frac{2\pi }{T}\exp\left(-\Omega_{mn}t\right)\right]+\\ \frac{\frac{2\pi }{T}\sin\left(\frac{2\pi t_{0}}{T}\right)}{\Omega_{mn}^{2}+4\pi^{2}/T^{2}}\left[\Omega_{mn}\cos\left(\frac{2\pi t}{T}\right)+\frac{2\pi }{T}\sin\left(\frac{2\pi t}{T}\right)-\Omega_{mn}\exp\left(-\Omega_{mn}t\right)\right] \end{array}$$

Additionally,

(27) $$\Omega_{mn}=\alpha \left(n^{2}+\frac{\nu_{m}}{W^{2}}+\frac{9}{4}\right)$$

### 3.3. Application to In Vivo Tomographic Measurements

In order to test its soundness, the model proposed by the present theoretical study was tested based on conditions and results reported by Linde et al. [27] and George et al. [25] by an ad hoc constructed MATLAB routine. Both these studies demonstrated that transmural perfusion ratio (TPR) (which is typically evaluated for a given myocardial slice segment as the local tomographic mean attenuation density at subendocardium divided by the mean attenuation density in the entire subepicardial layer) is considerably influenced by stenosis presence and severity and, therefore, can be a potentially strong marker of the hemodynamic impact of arteriosclerotic lesions. The analytical model proposed by the present study provides pressure and (from Darcy’s law) flow rate space–time distribution within a generic short-axis slice of the left ventricle. Therefore, it can be used to obtain a measure of regional transmural perfusion very close to TPR, as later specified. The typical diameter of myocardial capillaries is about 5–10 μm [13]. Thus, for this preliminary validating test, specific storage and hydraulic conductivity at the endocardial (purely capillary) level were estimated by analogy as specific storage and hydraulic conductivity characterizing clay soils saturated by water: $S_{s1}\approx 10^{-3}{\mathrm{ft}}^{-1}=10^{-3}/0.3048{\;m}^{-1}$, $K_{1}\approx 10^{-10}\;m/s$ [37]. Leaving parameters *q*_0*H*_ and *δ_H_* undetermined and free to be selected in such a way as to reproduce in both cases the values of the real basal hemodynamic parameters allowed for suitable calibration.

In their experimental investigation, Linde et al. [27] evaluated the relationship between the severity of coronary artery stenosis and the corresponding transmural perfusion ratio at rest and during adenosine stress by performing multidetector computed tomography (MDCT) on 200 symptomatic patients. Out of the 200 patients tested during the medical survey, diameter artery stenosis >50% was present in 49 (23 in the left anterior descending artery (LAD), 17 in the right coronary artery (RCA) and 9 in the circumflex artery (CX)). Note that, based on the functional segmentation reported by Cullen et al. [38], one half of the left ventricle shell is fed by LAD, three tenths are fed by RCA and one fifth is fed by CX.

In order to perform the comparison between predicted and observed TPR, the suitable value of mid diastole/mid systole healthy-control ventricle inflow *q*_0*H*_ and capillary transfer rate *δ_H_* was numerically searched for to match the main perfusion parameters in healthy/control conditions indicated by the authors: diastolic epicardial coronary pressure $p_{2}^{\prime }=p_{2}+p_{C}$ ranging between 112 and 155 mmHg for average heart period *T_H_* = 60/58 = 1.0345 s, and TPR ranging between 0.93 and 1.05 in the absence of stenosis. The result was *q*_0*H*_ = 2.5 × 10^−9^ m^2^/s and *δ_H_* = 3.0 N/m^2^s. During the survey, heart rate increased significantly from 58 ± 9 at rest to 72 ± 12 during stress, whereas systolic blood pressure did not change (133 ± 21 vs. 134 ± 22 mmHg). Stress conditions (active hyperemia) in the present study were then simulated by heart period *T* = 60/72 = 0.8333 s and vessels–tissue transfer rate given by:

(28) $$\delta =\frac{\delta_{H}T_{H}}{2\pi q_{0H}}\frac{\left[\left(2\pi -\Theta_{s}\right)+\xi \Theta_{s}\right]q_{0}}{T}$$

The assumption underlying Equation (28) is that actual capillary transfer rate and, consequently, metabolic oxygen consumption are directly proportional to actual heart frequency 1/*T* and circumferentially weighted ventricle inflow rate $\left(\left(2\pi -\Theta_{s}\right)+\xi \Theta_{s}\right)q_{0}.$

In the experimental study by George et al. [25], 40 symptomatic patients underwent adenosine-stress 64-row (*n* = 24) or 256-row (*n* = 16) detector computed tomography perfusion imaging (CTP) and computed tomography angiography (CTA). As for Linde et al. [27], the results were expressed in terms of (stress) TPR as a function of coronary occlusion degree, although nothing was said about the percentage of LAD, RCA and CX stenoses. The suitable value of the unknown parameters (*q*_0*H*_ = 2.7 × 10^−9^ m^2^/s and *δ_H_* = 2.5 N/m^2^s) was determined in such a way as to reproduce stress diastolic epicardial pressure ranging between 129 and 149 mmHg and healthy TPR ranging between 0.99 and 1.25. Since in both the medical surveys pharmacological stress was induced by adenosine administration, in the absence of indications about *T_H_* by George et al. [25], the ratio *T*/*T_H_* was assumed as coinciding with that reported by Linde et al. [27].

In both tests, the hyperemic non-stenotic coronary flow rate was not changed (*q*_0_ = *q*_0*H*_) based on the experimental findings by Momen et al. [39], which suggest that in healthy humans mild levels of stress can modulate coronary vascular tone by neural and/or direct vascular mechanisms. Finally, function ξ=ξ(%sten) was inferred from the curve of relative (stenotic/normal) flow reserve [40]. Note that, whereas in the medical survey adenosine stress occurred in patients who were already suffering from coronary diseases, in the model proposed by the present study coronary stenosis and metabolic acceleration occur instantaneously and simultaneously. Nevertheless, since ventricular pressure and flow rate are estimated at a regime diastolic peak (the first diastolic peak after two hours from the inception of the modified perfusion condition: *t_dp_* = *nT*/2 s, *n* = Int((2⋅3600)/(*T*/2)) +1), when the effect of the specific initial condition is already vanished, the two situations can reasonably be assimilated. Additionally, according to medical protocols, TPR was estimated by subdividing the ventricle wall into three main concentric layers (subendocardium, mid-myocardium and subepicardium) and by computing the ratio of local subendocardial flow rate to mean flow rate in the entire subepicardial layer. Table 1 shows the comparison between the average TPR estimated by Linde et al. [27] and the average TPR obtained by the present study, both at rest and during stress, and for stenosis degree lower and higher than 50%. Note that the TPR values estimated by the present study for stenosis degree higher than 50% were obtained by performing weighted average over LAD (Θ*_s_* = 180°), RCA (Θ*_s_* = 108°) and CX (Θ*_s_* = 72°) output. Table 2 shows the comparison between the average TPR during stress observed by George et al. [25] and that obtained by the present study by arithmetic mean performed over the corresponding LAD, RCA and CX output. Overall, the predictions of the theoretical model are very close to reality for both the medical surveys.

**Figure 4:**
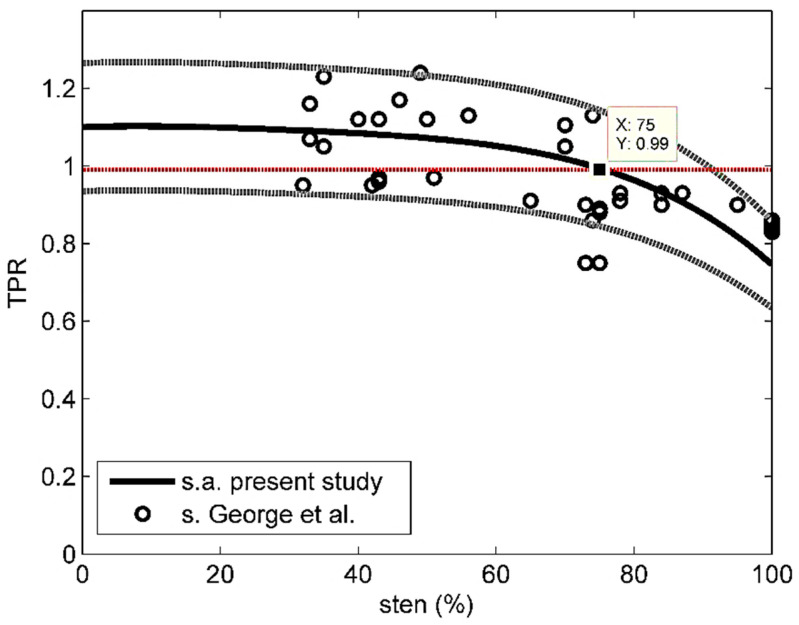
Comparison between stress TPR estimated by George et al. [25] and average stress TPR estimated by the present study; ‘s’ stands for stress and ‘s.a.’ for stress-average. Gray dotted lines indicate 85% confidence intervals.

Figure 4 shows the stress TPR data by George et al. [25] (*sten* > 30%) and the average stress curve obtained by the present study, along with the corresponding 85% confidence intervals. Only 2 of 35 experimental data fall outside those intervals. The analytical average stress curve was closely approximated by the following polynomial:

(29) $${\mathrm{TPR}}_{c}=-9\cdot 10^{-9}{sten}^{4}+10^{-6}{sten}^{3}-5.2\cdot 10^{-5}{sten}^{2}-0.00066\;sten+1.1$$

where subscript *c* stands for computed and 0 ≤ *sten* ≤ 100. The mean relative error evaluated based on Equation (29):

(30) $$err=\frac{1}{M}\sum_{i=1}^{M}\left|\frac{{TPR}_{ci}{-TPR}_{oi}}{{TPR}_{oi}}\right|$$

where subscript *o* stands for observed and *M* = 35, was 10%.

Note also that, based on Figure 4 and the analytical average stress curve, the TPR-threshold discriminating normal from anomalous perfusion (TPR = 0.99 after [25]) would correspond to about 75% stenosis, which is very close to what is commonly recognized by the medical community as the critical degree of coronary occlusion (80%).

According to the outcome of the present theoretical model, in conditions of normal perfusion and during diastole, the distribution of pressure within the ventricular arterial network is circumferentially homogeneous, with the higher and the lower values located in the subepicardial and subendocardial layers, respectively. As a matter of fact, based on the effective stress–interstitial pressure relationship $\sigma =\bar{\sigma }+p$, and for a given value of external overload *σ*, a smaller blood pressure *p* corresponds to a larger muscular stress $\bar{\sigma }$ and vice versa. Thus, the results are consistent with the notion that the effect of the extravascular compressive forces (intracavitary ventricle pressure and intramyocardial pressure) are stronger at subendocardium [41]. Conversely, consistently with the experimentally detected healthy TPR larger than 1, the higher and the lower values of the purely radial **U** are obtained at subendocardium and at subepicardium, respectively. Additionally, according to Equation (2), at the peak of systole (*t*_sp_ = *nT*) the ventricular stress state is practically reduced to the only muscular component and *p* ≅ 0 mmHg (not shown).

**Figure 5:**
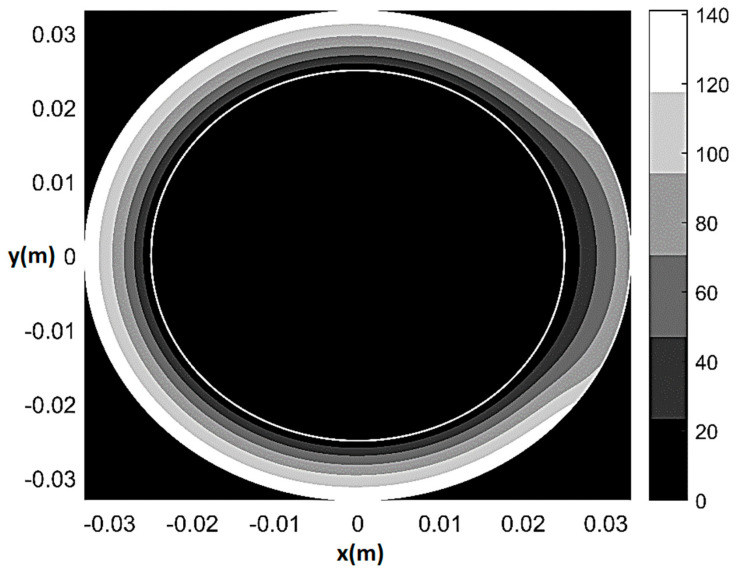
Pressure contour plot at rest for 80% CX stenosis (*q*_0_ = *q*_0*H*_ = 2.7 × 10^−9^ m^2^/s, *T*/*T_H_* = 1, *ξ* = 0.55, Θ*_s_* = 72°, *δ*/*δ_H_* = 1). Pressure values in mmHg.

**Figure 6:**
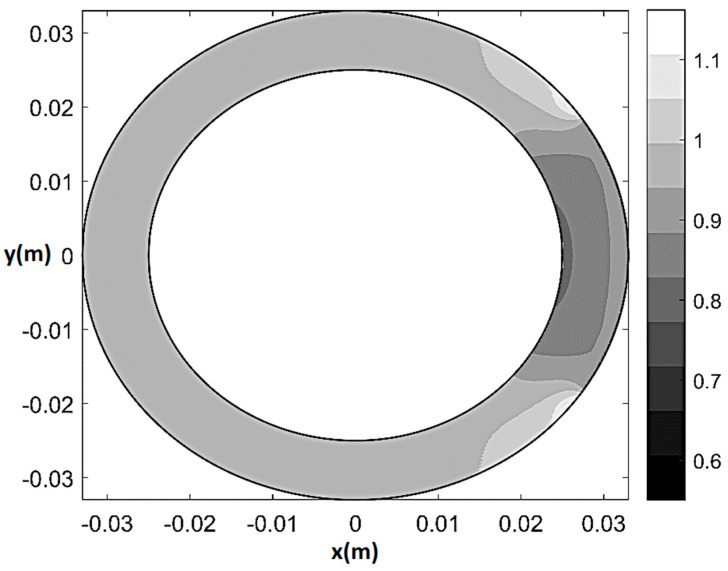
Normalized flow rate contour plot (*U*/*U_H_*) at rest for 80% CX stenosis (*q*_0_ = *q*_0*H*_ = 2.7 × 10^−9^ m^2^/s, *T*/*T_H_* = 1, *ξ* = 0.55, Θ*_s_* = 72°, *δ*/*δ_H_* = 1).

As an example of the model performance in terms of prediction of physiological and pathological pattern of myocardial perfusion, Figure 5 and Figure 6 respectively show pressure and normalized flow rate *U_H_*/*U*_max*H*_ contour plots (where U=|U| and *U*_max*H*_ indicates the maximum *U* in healthy-control conditions) at rest in the presence of 80% CX stenosis (*q*_0_ = *q*_0*H*_ = 2.7 × 10^−9^ m^2^/s, *T*/*T_H_* = 1, *ξ* = 0.55, Θ*_s_* = 72°, *δ*/*δ_H_* = 1). The maps are built on a 501 × 501 Cartesian computational grid ($r=\sqrt{x^{2}+y^{2}\;}\;\theta =\tan^{-1}\left(y/x\right)\;$) based on the superposition of 50 Fourier harmonics. As one can see from Figure 5, in the presence of stenosis the lower isobars start to migrate toward the underperfused subepicardial region, causing the local decrease in vessels’ pressure. Additionally, a trapezoidal region of mild flow rate reduction (0.85 ≤ *U*/*U_H_* ≤ 0.95) surrounding a small convex sickle-like region of maximum flow rate reduction (0.75 ≤ *U*/*U_H_* ≤ 0.85) appears at the subendocardium in correspondence of the underperfused sector (Figure 6). This result may represent a possible fluid-mechanically-based answer to the question about the reasons of the greater vulnerability of subendocardium to ischemia, which has recently been posed in literature [42]. Specifically, the mathematical model shows that, in the case of non-homogeneous epicardial blood supply, the activation of a circumferential flux $U_{\theta }=-\left(K/r\gamma \right)\partial p/\partial \theta$ determines a non-uniform side diffusion that has its peak-values (same order of magnitude of the radial $U_{r}=-\left(K/\gamma \right)\partial p/\partial r$) at the epicardial extremes of the underperfused ventricle sector. Thus, whereas at subendocardium the flow remains essentially radial and markedly affected by the sectorial stenotic perfusion, at subepicardium it is enhanced by a strong circumferential component, which conveys fresh blood entering the ventricle at the epicardial contour of the underperfused sector neighboring zones.

**Figure 7:**
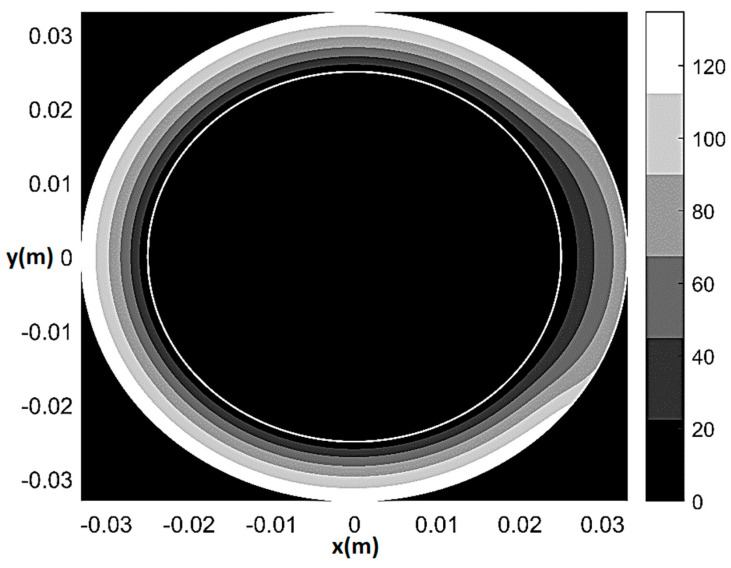
Pressure contour plot under stress for 80% CX stenosis (*q*_0_ = *q*_0*H*_ = 2.7 × 10^−9^ m^2^/s, *T*/*T_H_* = 0.8055, *ξ* = 0.55, Θ*_s_* = 72°, *δ* given by (28)). Pressure values in mmHg.

**Figure 8:**
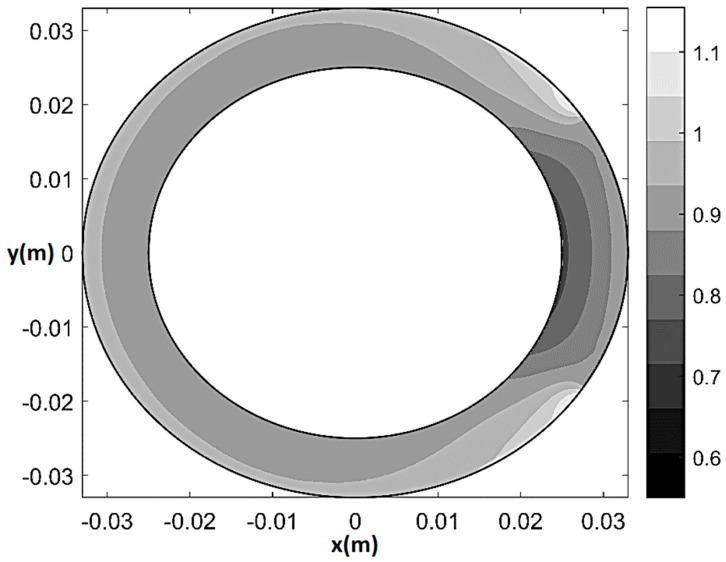
Normalized flow rate (*U*/*U_H_*) contour plot under stress for 80% CX stenosis (*q*_0_ = *q*_0*H*_ = 2.7 × 10^−9^ m^2^/s, *T*/*T_H_* = 0.8055, *ξ* = 0.55, Θ*_s_* = 72°, *δ* given by (28)).

Finally, Figure 7 and Figure 8 show pressure and normalized flow rate contour plot during stress for 80% CX stenosis (*q*_0_ = *q*_0*H*_ = 2.7 × 10^−9^ m^2^/s, *T/T_H_* = 0.8055, *ξ* = 0.55, Θ*_s_* = 72°, *δ* given by (28)). The main difference as compared to the rest 80%-stenosis case was detected in terms of flow rate distribution. Specifically, the mild underperfusion (5–15% reduction in flow rate) now affected the whole myocardial shell, while the convex sickle-like region characterized by 15–25% perfusion reduction was much larger than at rest and affected subendocardium as well as mid-myocardium. Additionally, a non-negligible decrease in blood flow also affected remote myocardium (π/2≤θ≤3π/2).

It should be emphasized that, if stenosis were not removed, even the simply ischemic zones, which are characterized by a pathologic though non-immediately critical reduction in pressure and oxygen supply as compared to healthy conditions, would be at risk of necrosis in the long period. Thus, the pattern of isobars and normalized isotachs obtained by the present model, which exhibits a ubiquitous negative trend towards the deepest ventricle layers, may explain the myocardium necrosis time-progression from endocardium to epicardium as a “wave front phenomenon” [1]. Finally, the model cannot formally account for the variation of conductivity and storativity due to the death of ventricle cells. Thus, its validity is restricted to the prenecrosis period, which nevertheless is the crucial timescale for medical interventions and reperfusion attempts.

## 4. Conclusions

An analytical model is for the first time proposed for the interpretation of the fluid-mechanical processes governing left ventricle wall perfusion in healthy and ischemic conditions. The model is based on the confined porous media theory and the use of a suitable diffusion equation, which accounts for the typical swelling–drainage alternation characterizing the diastolic–systolic phases by a volumetric specific storage, and for the arterial/venous network exchange by a suitable sink term. The ventricle wall, which physically represents the flow domain to be modelled, is assimilated to a porous semi-ellipsoidal shell characterized by decreasing permeability and horizontal short-axis. The analysis is restricted to the portion of arterial network contained in a unit-height, approximately cylindrical short-axis element of the ellipsoidal shell, fed by an axial-symmetric (healthy perfusion) or a non-axial-symmetric (stenotic perfusion) blood flow rate. The presence of an epicardial coronary stenosis of variable severity determines a proportionate reduction in pressure and flow rate within a more or less extended ventricle sector. The lowest isobars progressively migrate from the endocardial to the epicardial boundary and the subendocardial microvessels tend to approach their critical closing pressure. The stenotic percentage reduction of blood flow rate as compared to healthy-control conditions is larger at subendocardium even at rest. That result may constitute a possible fluid-mechanically-based answer to the question of the greater vulnerability of the internal ventricle layers to ischemia.

The comparison in terms of rest and stress transmural perfusion ratio with the MDCT measurements performed by two independent medical surveys proved to be quite satisfactory, demonstrating the predictive ability of the model in assessing that fundamental hemodynamic indicator.

Finally, since the proposed model is fully analytical and yields an exact solution for the local time-dependent myocardial pressure and flow rate, it may be particularly useful for the application of inverse methods aimed at estimating the ischemic perfusion parameters (i.e., location and severity of coronary stenoses) that better fit non-invasive downstream tomographic measurements.

It should be emphasized that the model in itself cannot account for the variation of conductivity and storativity due to the death of ventricle cells. Thus, its validity is restricted to subocclusive (eventually asymptomatic) stenoses or to the prenecrosis period, which nevertheless is the crucial timescale for medical interventions and reperfusion attempts.

## 5. Limitations of the Model and Clinical Applicability Perspectives

The present study proposes a two-dimensional, fully analytical fluid-mechanical model of healthy and ischemic perfusion of the left ventricle wall based on saturated porous media flow theory. While the solution of the resulting boundary value problems in cylindrical coordinates is exact and does not suffer any approximation (it is expressed as rapidly converging blood pressure and flow rate Fourier series), the ventricle scheme and its physiopathological function are necessarily simplified. First, it is assumed that the ventricle wall is a perfectly semiellipsoidal shell characterized by a sufficiently weak curvature in the longitudinal planes that allows for a local cylindrical approximation and, therefore, a local two-dimensional schematization. Second, it is assumed that diastolic swelling and systolic drainage are dominated by radial blood flux from epicardium to endocardium and vice versa: in this case, the short-axis slices coupling determined by the longitudinal flow paths can be considered as a high-order effect. Third, it is assumed that ventricular wall hemodynamics is essentially governed by the cyclic (globally conservative) rearrangement of the volumes occupied by blood-filled vessels and muscular fibers, and that ventricle torsion and wall thickness variations only play a marginal role. Fourth, the periodic ventricle wall feeding is schematized as a single harmonic characterized by the effective maximum amplitude and the effective heart period. This means that, in the present version, the model does not account for the possible effects of a periodically random signal. Finally, it should be emphasized that it cannot account for the variation of conductivity and storativity due to the death of ventricle cells related to tissue post-infarction necrosis. Thus, its validity is restricted to subocclusive (eventually asymptomatic) stenoses or to the prenecrosis period, which nevertheless is the crucial timescale for medical interventions and reperfusion attempts.

The scope of this preliminary investigation was to show that: (1) saturated porous media flow theory is indeed a suitable framework for investigating ventricle wall hemodynamics; (2) the basic model, which is characterized by a reduced number of degrees of freedom, allows for an exact analytical solution and, therefore, for a straightforward implementation of inverse solution techniques aimed at assessing parameters *ξ* (severity of the stenosis) and Θ*_s_* (location of the stenosis), is at the same time able to grasp the main perfusion mechanisms and quantitatively agrees with the results of clinical in vivo observations.

Future research developments will include model generalizations like the analysis of three-dimensional effects and cardiac signal irregularity, possibly keeping the computational complexity below an upper limit that makes it still compatible with inverse methods implementation. Given for granted the possibility to assess the evolving ventricle wall flow rate distribution by imaging techniques, once fully tested, the model could be used to detect the presence, location and severity of subocclusive and/or asymptomatic coronary stenoses without resorting to invasive and potentially risky medical procedures.

Already in the present version, the model could constitute a useful interpretative support to improve the comprehension of the basic hemodynamic mechanisms leading to the most common cardiac diseases.

## Figures and Tables

**Table 1:** Comparison between average TPR estimated by Linde et al. [27] and by the present study. SD stands for standard deviation. The numbers in parenthesis indicate the corresponding ranges.

TPR	Linde et al. [27]	Present Study
Average at rest *sten* < 50%	0.955 SD = 0.081 (0.874–1.036)	1.04
Average during stress *sten* < 50%	0.942 SD = 0.047 (0.895–0.989)	0.963
Average at rest *sten* > 50% LAD(23), RCA(17), CX(9)	0.943 SD = 0.087 (0.856–1.03)	0.913
Average during stress *sten* > 50% LAD(23), RCA(17), CX(9)	0.88 SD = 0.088 (0.792–0.968)	0.844

**Table 2:** Comparison between average stress TPR estimated by George et al. [25] and by the present study. SD stands for standard deviation. The numbers in parenthesis indicate the corresponding ranges.

TPR	George et al. [25]	Present Study
Average during stress *sten* = 0%	1.12 SD = 0.13 (0.99–1.25)	1.09
Average during stress *sten* 30–49%	1.09 SD = 0.11 (0.9–1.12)	1.076
Average during stress *sten* 50–69%	1.06 SD = 0.14(0.92–1.2)	1.046
Average during stress *sten* 70–100%	0.91 SD = 0.1 (0.81–1.01)	0.916

## Data Availability

All available data are included in the paper.

## Abbreviations

σ	total vertical stress within the ventricle wall
$\bar{\sigma }$	vertical stress adsorbed by the muscular fibers within the ventricle wall
p	blood transmural pressure
U	Darcy flow rate vector
K	hydraulic conductivity
γ	blood specific weight
r	radial coordinate
θ	angular coordinate
z	vertical coordinate
$S_{s}$	specific storage
t	time
$\tilde{q}$	volume of blood leaving the arterial network per unit time and unit ventricle volume
ω	ventricle/porous medium porosity
$\varepsilon_{s}$	coefficient of elasticity of the muscular component
ε	bulk coefficient of elasticity
$\beta_{f}$	blood coefficient of compressibility
$d_{v}$	pore/vessel average diameter
$p_{H}$	healthy transmural pressure that exceeds capillary pressure
$p_{c}$	capillary pressure
s	ventricle wall thickness
$q_{H}$	healthy amplitude of pulsatile flow rate
$q_{0H}$	healthy mid diastole/mid systole flow rate
$T_{H}$	healthy cardiac cycle period
$q_{s}$	stenotic amplitude of pulsatile flow rate
$q_{0}$	stenotic mid diastole/mid systole flow rate
$\Theta_{s}$	angle subtended by the stenotic ventricle sector
ξ	coefficient of reduction of flow rate in stenotic conditions
T	stenotic cardiac cycle period
TPR	transmural perfusion ratio
sten	percentage of stenosis
err	relative error in the assessment of TPR
$U_{r}$	radial component of Darcy velocity
$U_{\theta }$	angular component of Darcy velocity
U	magnitude of Darcy velocity
$U_{H}$	healthy magnitude of Darcy velocity
$U_{maxH}$	maximum healthy magnitude of Darcy velocity
